# Temporal Bone Fracture Causing Superior Semicircular Canal Dehiscence

**DOI:** 10.1155/2014/817291

**Published:** 2014-09-10

**Authors:** Kevin A. Peng, Sameer Ahmed, Isaac Yang, Quinton Gopen

**Affiliations:** ^1^Department of Head and Neck Surgery, David Geffen School of Medicine at the University of California, Los Angeles, 10833 Le Conte Avenue, CHS 62-132, Los Angeles, CA 90095, USA; ^2^Department of Neurosurgery, David Geffen School of Medicine at the University of California, Los Angeles, CA 90095, USA

## Abstract

*Importance*. Superior semicircular canal dehiscence (SCD) is a third window lesion of the inner ear causing symptoms of vertigo, autophony, tinnitus, and hearing loss. A “two-hit” hypothesis has traditionally been proposed, whereby thinly developed bone overlying the superior canal is disrupted by a sudden change in intracranial pressure. Although the symptoms of SCD may be precipitated by head injury, no previous reports have described a temporal bone fracture directly causing SCD. *Observations*. Two patients sustained temporal bone fractures after closed head trauma, and developed unilateral otologic symptoms consistent with SCD. In each instance, computed tomography imaging revealed fractures extending through the bony roof of the superior semicircular canal. *Conclusions and Relevance*. Temporal bone fractures, which are largely treated nonoperatively, have not previously been reported to cause SCD. As it is a potentially treatable entity, SCD resulting from temporal bone fracture must be recognized as a possibility and diagnosed promptly if present.

## 1. Introduction

Superior semicircular canal dehiscence syndrome (SCD) has attracted significant attention since first being described by Minor et al. in 1998 [1]. In this condition, the bone overlying the superior semicircular canal is dehiscent, resulting in a “third window lesion” that produces variable amounts of vestibular and audiologic dysfunction. Loud sounds or changes in middle ear pressure (the Tullio phenomenon and the Hennebert sign, resp.) cause motion of perilymph and endolymph between the vestibule and dehiscent superior semicircular canal [1–3]. The resulting symptoms of vertigo, autophony, tinnitus, and hearing loss present in varying degrees [4]. Due to the variety of potential symptoms, diagnosis of SCD may be delayed in favor of confounding conditions, including Meniere's disease, otosclerosis, and perilymphatic fistula, among others. In addition to conductive hearing loss, SCD has also been implicated in mixed and profound sensorineural losses [5]. SCD has also been described in children and in postpartum females [6, 7].

It is commonly held that thin or absent bone overlying the superior semicircular canal in the middle cranial fossa predisposes patients to the syndrome. Carey et al. performed an analysis of 1000 temporal bones from 596 patients and concluded that the bone overlying the superior canal is thin at birth for the vast majority of humans but progressively thickens until three years of age. Approximately 2% of adults have dehiscent or extremely thin bone overlying the superior semicircular canal (<0.1 mm; normal, 0.96 ± 0.61 mm). However, dehiscent or extremely thin bone alone over the superior canal does not definitively predict symptoms of SCD [8]. Approximately half of the patients with SCD in Minor's study from 2000 had an antecedent event of closed head trauma or barotrauma causing sudden changes in intracranial pressure [2]. These findings led Carey et al. to postulate that a “two-hit” hypothesis may account for producing the classic symptoms seen in SCD: the “first hit” is the thin bone that fails to thicken postinfancy, while the “second hit” is a derangement, including head trauma, that disrupts thin bone or stable dura over the superior canal, resulting in abnormal motion of the endolymph or perilymph [8].

While minor head trauma leading to changes in intracranial pressure has been implicated as an etiology of SCD in previous studies, no previous work has explored the causality behind temporal bone fractures precipitating SCD. We present the first series of two patients who, following significant head trauma, suffered temporal bone fractures in association with superior semicircular canal dehiscence. A review of the literature yields a discussion about head trauma in superior semicircular canal dehiscence.

## 2. Case Reports

### 2.1. Case 1

**Figure 1:**
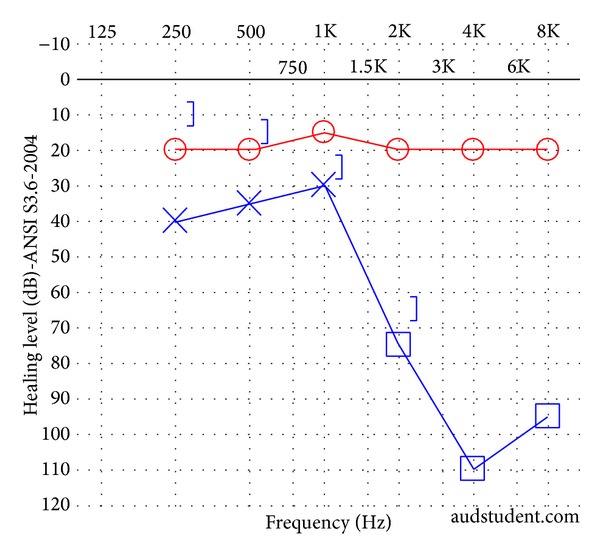
Audiogram of patient in Case 1. A left mixed hearing loss is present.

**Figure 2:**
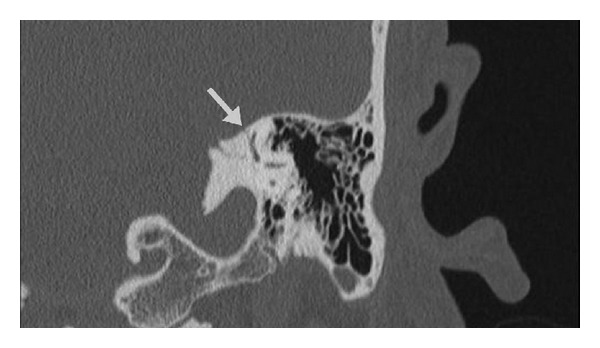
Computed tomography (CT) imaging of the patient in Case 1. Coronal section demonstrates a left temporal bone fracture (arrow) traversing the bony superior semicircular canal.

A 37-year-old previously healthy male presented as a code trauma after sustaining blunt force injury to the head and extremities with a baseball bat. His initial otologic complaints were dizziness, left-sided tinnitus, and left-sided hearing loss. Otoscopic examination revealed a left-sided hemotympanum. At 512 Hz, the Weber test lateralized to the fractured side, while the Rinne test demonstrated bone conduction louder than air conduction on the fractured side. Facial nerve function was symmetric and intact bilaterally (House-Brackmann grade I/VI). On follow-up visits to the clinic, he continued to have left-sided pulsatile tinnitus and hearing loss along with hyperacusis and disequilibrium. An audiogram was performed (Figure 1), demonstrating normal hearing on the right and a mild-to-severe mixed hearing loss on the left with an air-bone gap of 30 dB in the lower frequencies. Speech reception threshold (SRT) was 15 dB on the right and 35 dB on the left. Stapedial reflexes were intact. Vestibular evoked myogenic potential (VEMP) testing was not performed. Computed tomography (CT) was performed, demonstrating a left mixed longitudinal and transverse fracture extending into the superior semicircular canal but sparing the facial nerve and cochlea (Figure 2). The ossicles appeared to be intact and in continuity.

Given the concern for a traumatic perilymphatic fistula and his persistent symptoms despite observation, the patient elected to undergo surgical management. The patient underwent a middle ear exploration; a normal ossicular chain was identified, and there was no sign of an oval or round window rupture. Postoperatively, the patient had no change in his symptoms, corroborating the persistence of a third window lesion.

### 2.2. Case 2

A 28-year-old female patient with no chronic medical problems was struck by a bus while walking as a pedestrian on a city street. She suffered bilateral frontal contusions and a large subdural hematoma that required an urgent craniectomy. The patient was stabilized, monitored in the hospital, and then discharged to specialized neurorehabilitation. During her rehabilitation, she complained of difficulty hearing from her right ear, right-sided pulsatile tinnitus, and disequilibrium. Of note, the patient reported a history of a right congenital cholesteatoma for which she underwent two tympanomastoidectomies as a child, with no reported involvement of the ossicular chain. Prior to the trauma, she had no audiologic or vestibular symptoms.

**Figure 3:**
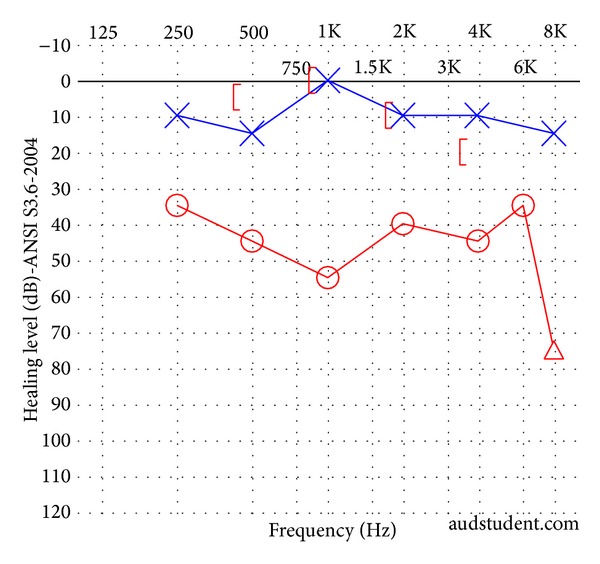
Audiogram of patient in Case 2 following trauma but prior to middle ear exploration. A right mixed hearing loss is present. This patient presented radiographically with both superior semicircular canal dehiscence and an ipsilateral incudomalleolar joint dislocation.

**Figure 4:**
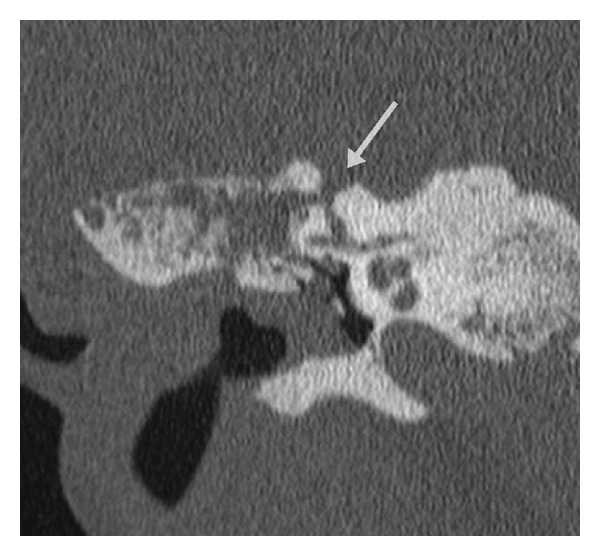
CT imaging of the patient in Case 2. Coronal section demonstrates a right temporal bone fracture (arrow) traversing the bony superior semicircular canal.

Otoscopic examination was within normal limits, with no cholesteatoma identified. Facial nerve function was symmetric and intact bilaterally (House-Brackmann grade I/VI). Audiologic examination showed normal hearing in the left ear and a mild to severe conductive hearing loss across all frequencies in the right ear (Figure 3). VEMP testing was not performed. CT imaging revealed a right mixed longitudinal and transverse right temporal bone fracture that spared the cochlea. The bone overlying the superior semicircular canal was noted to be thin and the fracture extended into this area (Figure 4). The incudomalleolar joint was also dislocated, presumably from the trauma. The patient was brought to the operating room to address the noted ossicular discontinuity. During the same anesthetic, she also underwent a right temporoparietal cranioplasty by the neurosurgery team.

**Figure 5:**
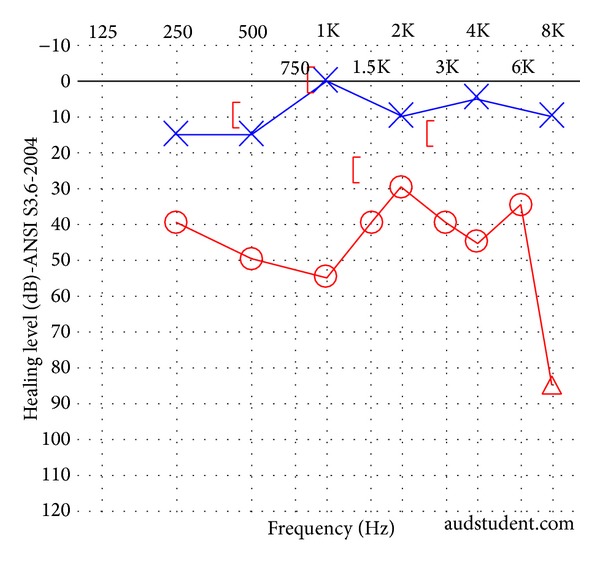
Audiogram of patient in Case 2 following right middle ear exploration. The mixed hearing loss persists, possibly suggesting an uncorrected third window lesion.

Intraoperatively, ossicular discontinuity was confirmed, and no perilymphatic fistula was noted. The stapes superstructure was intact and the footplate was mobile. She was rehabilitated with a partial ossicular reconstruction prosthesis. The fracture into the superior canal was intentionally not explored, as the patient did not complain of debilitating vestibular symptoms. Postoperatively, the patient claimed that her hearing had improved to her preaccident baseline, and she had no signs or symptoms of vertigo, dizziness, autophony, or tinnitus. A postoperative audiogram showed a persistent 40 dB air-bone gap in the low- to mid-frequencies (Figure 5), consistent with a persistent conductive loss possibly suggestive of a persistent third window lesion.

## 3. Discussion

Previous research in etiologic factors of SCD has strongly suggested the role of underdeveloped bone overlying the superior semicircular canal predisposing patients to the symptoms of SCD after a “second hit” [8]. A radiologic study done by Nadgir et al. showed that the incidence of dehiscence or thin bone overlying the superior semicircular canal, or the “first hit,” increases with increasing age, indicating that SCD is most likely an acquired condition over a congenital one [9]. However, previous reports from Lee et al. have demonstrated that this condition can be seen in children as well [6].

The “second hit” usually refers to trauma causing an unroofing of the thin bony covering of the semicircular canal; for example, Watters et al. reported two postpartum patients found to have SCD, with the presumed increased intracranial pressure during childbirth serving as the “second hit.” In their study, they further reported that, on meta-analysis, 48% of patients undergoing surgical correction of SCD recalled some form of a “second hit” [7]. Minor's study from 2005 examined nonsurgical and surgical cases of SCD, with 23% of patients noting a specific occurrence, including head trauma or changes in middle ear/intracranial pressure, that precipitated audiologic and/or vestibular symptoms [10].

Here, we present in two patients the previously unreported association of temporal bone fracture directly causing SCD via fracture through the bony roof of the superior semicircular canal. While previous reports have suggested that radiological evidence of SCD is found in approximately 9% of all temporal bone CT imaging, [11] the dehiscences observed in the current two presentations each involved a fracture grossly violating the bone of the superior semicircular canal rather than simply a thinning of the overlying bone. In both cases, this resulted in varying degrees of vestibular and audiologic symptoms. Perilymphatic fistula, an important differential diagnostic consideration, was ruled out intraoperatively in both cases. Furthermore, in the second case, middle ear surgery with successful rehabilitation of the ossicular discontinuity with a PORP did not correct the patient's conductive hearing loss; this is consistent with prior literature describing persistent conductive loss after middle ear surgery as suggestive of a third window lesion [12].

## 4. Conclusion

These two cases reemphasize that following stabilization of a patient with blunt or penetrating temporal bone trauma, symptoms suggestive of a third window lesion must be systematically evaluated, and a perilymphatic fistula must be ruled out as a differential diagnosis. SCD resulting from temporal bone fracture is a treatable entity that may offer resolution of symptoms. As otolaryngologists, we must remain cognizant that temporal bone trauma, changes in intracranial pressure, and temporal bone fractures, as reported here, may all precipitate SCD.
